# Serum exosomal microRNA-1258 may as a novel biomarker for the diagnosis of acute exacerbations of chronic obstructive pulmonary disease

**DOI:** 10.1038/s41598-023-45592-4

**Published:** 2023-10-26

**Authors:** Fei Wang, Boxin Yang, Jiao Qiao, Linlu Bai, Zijing Li, Wenyuan Sun, Qi Liu, Shuo Yang, Liyan Cui

**Affiliations:** 1 Department of Respiratory and Critical Care Medicine, Peking University Third Hospital, 49 North Garden Road, Haidian District, Beijing, 100191 People’s Republic of China; 2 Department of Laboratory Medicine, Peking University Third Hospital, 49 North Garden Road, Haidian District, Beijing, 100191 People’s Republic of China; 3 Peking University, No.5 Yiheyuan Road Haidian District, Beijing, People’s Republic of China

**Keywords:** Molecular biology, Biomarkers, Molecular medicine

## Abstract

Acute exacerbation chronic obstructive pulmonary disease (AECOPD) has a high mortality rate. However, there is no efficiency biomarker for diagnosing AECOPD. The purpose of this study was to find biomarkers that can quickly and accurately diagnose AECOPD.45 normal controls (NC), 42 patients with stable COPD (SCOPD), and 66 patients with AECOPD were enrolled in our study. Serum exosomes were isolated by ultracentrifuge and verified by morphology and specific biomarkers. Fluorescent quantitation polymerase chain reaction (qRT-PCR) was used to detect the expression of micro RNAs (miRNAs), including miR-660-5p, miR-1258, miR-182-3p, miR-148a-3p, miR-27a-5p and miR-497-5p in serum exosomes and serum. Logistic regression and machine learning methods were used to constructed the diagnostic models of AECOPD. The levels of miR-1258 in the patients with AECOPD were higher than other groups (*p* < 0.001). The ability of exosomal miR-1258 (AUC = 0.851) to identify AECOPD from SCOPD was superior to other biomarkers, and the combination of exosomal miR-1258 and NLR can increase the AUC to 0.944, with a sensitivity of 81.82%, and specificity of 97.62%. The cross-validation of the models displayed that the logistic regression model based on exosomal miR-1258, NLR and neutrophil count had the best accuracy (0.880) in diagnosing AECOPD from SCOPD. The three most correlated biomarkers with serum exosome miR-1258 were neutrophil count (r = 0.57, *p* < 0.001), WBC (r = 0.50, *p* < 0.001) and serum miR-1258 (r = 0.33, *p* < 0.001). In conclusion, serum exosomal miR-1258 is associated with inflammation, and can be used as a valuable and reliable biomarker for the diagnosis of AECOPD, and the establishment of diagnostic model based on miR-1258, NLR and neutrophils count can help to improving the accuracy of AECOPD diagnosis.

## Introduction

Acute exacerbations of chronic obstructive pulmonary disease (AECOPD) can lead to an accelerated decline in lung function, quality of life decreased and worsening long-term prognosis of patients^1^, and is the major cause of COPD-related morbidity including cardiovascular and cerebrovascular diseases^2,3^. Currently, the AECOPD diagnosis mainly relies on symptoms, however, since many complications present similar symptoms and the clinical presentation of AECOPD is heterogeneous, it’s difficult for physicians to identify acute exacerbations accurately in the first place based on symptoms alone. Therefore, exploring specific biomarkers for AECOPD is vital for improving the prognosis of patients and reducing the risk of death.

Some biomarkers including C-reactive protein (CRP) and procalcitonin (PCT) have been used as auxiliary diagnosis of AECOPD caused by bacterial infection^4,5^, regardless of the fact that bacterial infection accounts for only about 55% of all AECOPD causes, the accuracy of both in diagnosing bacterial infection is not optimal^6^.

Micro RNAs (miRNAs) is a conserved non-coding endogenous single-stranded RNA composed of 19–23 nucleotides. As a negative regulator, miRNAs inhibit candidate gene expression by binding to the 3′-untranslated region (UTR) of messenger RNA (mRNA), leading to mRNA degradation or translation inhibition^7^. MiRNAs play a vital role in biological processes such as cell differentiation, proliferation, metabolism, aging, and apoptosis^8^, therefore they have been widely studied as alternative biomarkers for diagnostic and prognostic purposes in various diseases, such as cancers, metabolic disorders, cardiovascular disease, drug induced liver injury and COPD^9^. Previous studies have recognized numbers of miRNAs that may have regulatory functions in the progression of COPD, such as miR-660-5p, miR-1258, miR-182-3p, miR-148a-3p, miR-27a-5p and miR-497-5p, but they have not been proven in clinic and the role in assisting the diagnosis of AECOPD remains unclear.

Serum exosomes are small nano-sized vesicles released into extracellular space after fusion of several multivesicular endosomes, which contain enriched amounts of specific surface markers, regulatory proteins, mRNA and miRNAs^3,10^. Cell-derived exosomes mediated cell-to-cell communication and immunoregulatory functions^8,11,12^. There is growing evidence that serum exosomes are enriched in serum, and their composition changes in disease state compare to normal physiological conditions, emphasizing the role of serum exosomes as novel circulating biomarkers^13^.

This study was designed to evaluate the role of the miRNAs,including miR-660-5p, miR-1258, miR-182-3p, miR-148b-3p, miR-27a-5p and miR-497-5p in the diagnosis, distinguishing the subtypes and severity of AECOPD, and to further establish prediction models based on miRNAs and other inflammatory factors through mathematical methods.

## Methods

### Subjects enrollment

From December, 2019 to June, 2021, 66 patients presenting for AECOPD were admitted to the Respiratory Department in Peking University Third Hospital as well as 45 SCOPD patients and 42 healthy subjects were enrolled. According to the Global Initiative for Chronic Obstructive Lung Disease (GOLD) classification^14^, inclusion criteria of SCOPD was: (1) a confirmed diagnosis of COPD with post-bronchodilator forced expiratory volume in 1 s (FEV 1) ≤ 80% of the predicted normal value and FEV 1 /forced vital capacity (FVC) ≤ 0.7, consistent with GOLD stage II–IV disease; (2) current or ex-smoker with smoking history ≥ 10 pack years; and (3) age ≥ 40 years. Patients with cystic fibrosis, tuberculosis, asthma, bronchiectasis, α-1 antitrypsin deficiency, history of lung surgery and lung cancers or other malignant tumors were excluded. AECOPD was defined as an event in the natural course of the disease characterized by acute changes in clinical symptoms beyond normal day-to-day variation, resulting in additional therapy. Specifically, AECOPD patients had at least two of three complications (sputum volume, purulence, dyspnea); or had a worsening of at least one major symptom (cold, nasal discharge and/or nasal congestion, wheeze and sore throat) and one minor symptom (cough and fever (oral temperature > 37.5 °C) without other causes)^15^. Other conditions that may cause these symptoms are excluded, including cardiac insufficiency, pulmonary embolism, pneumothorax, pleural effusion and pneumonia. This study was approved by Peking University Third Hospital Medical Science Research Ethics Committee (MR-11–22-001,995).The written informed consent from the patients is not required in the current study as the residual serum samples were used for our experiment after the laboratory tests,and the remains were useless for patient.The ethics committee granted an exemption for informed consent.

### Sample size calculation

According to the pre-experimental results, we used PASS 16.0 software (PASS Software, Amsterdam,Netherlands) to calculate the sample size. A sample of 41 patients with SCOPD and 41 patients with AECOPD, achieves 81% power to detect a difference of 0.15 between the AUC under the null hypothesis of 0.70 and an AUC under the alternative hypothesis of 0.85 using a two-sided z-test at a significance level of 0.05. In order to achieve better prediction effect, we enrolled a sample size of more than 41 in each group, ultimately including 66 patients with AECOPD, 45 with SCOPD, and 42 healthy controls.

### Clinical data and blood sample collection

Demographic data, tobacco exposure history, underlying diseases, comorbid conditions, symptoms, exacerbation frequency in the previous year of the subjects were recorded and recruited. All patients underwent high-resolution computed tomography (HRCT) scans within 7 days before hospitalization and emphysema were documented. Blood samples were collected for laboratory tests within 24 h of admission, placed in the vacuum tubes containing coagulant and separating gel (SEKISUI, Osaka, Japan), and immediately centrifuged at 3000 *g* for 10 min. The serum was isolated into Eppendorf tubes and stored at − 80 °C until analysis. CRP and lactate dehydrogenase (LDH) were tested by latex immunoturbidimetric assay kit on Beckman Coulter AU2700 (Beckman Coulter, California, America), PCT was detected by electrochemiluminescence assay on a Roche E401 (Roche Diagnostics, Mannheim, Germany).

Complete blood counts and leukocyte differential counts were detected by Sysmex XN 9000 (Sysmex Corporation, Kobe, Japan) within 2 h using EDTA anticoagulated blood samples.

### Harvest and isolate of serum exosomes

The serum exosomes^16–18^ were isolated using supercentrifugation (L-90KL-80XP, Beckman Coulter, California, America). Briefly, the serum samples were first centrifuged at 2000 g for 30 min to remove died cells and debris, then centrifuged at 100,000 *g* for 70 min, supernatant was discarded. The sediment was washed by 1 ml phosphate-buffered saline (PBS) and were centrifuged at 100,000 *g* for 70 min^19^, supernatant was discarded and the remaining was mainly serum exosomes. Finally, serum exosomes were resuspended with 200μL PBS for identification and RNA extraction.

### Identification of serum exosomes

*Transmission electron microscopy (TEM)* The exosome pellets were fixed in 3% (w/v) glutaraldehyde and 2% paraformaldehyde in cacodylate buffer, and loaded to copper grids that coated with formvar. After that, copper grids were washed, contrasted in 2% uranyl acetate, and dried, then examine by TEM (Morgagni 268D, Philips, Holland).

*Western blot* The specific exosomes surface markers, including CD63 (Abcam, Cambridge, British, Catalog number:ab134045), TSG101(Abcam, Cambridge, British, Catalog number:ab125011), and ALIX(Abcam, Cambridge, British, Catalog number:ab275377) were detected. According to routine protocols, the specific markers antibodies (Santa Cruz Biotechnology, California, America) (1:1000) and secondary antibodies (Cell Signaling Technology, Boston, America. Catalog number: 7074) (1:10,000) were incubated with membranes respectively and proteins were exposed by molecular imaging system (BIO-RAD, California, America).

*Zeta sizer Nano tracking analysis* Size distribution of serum exosomes was detected by dynamic light scattering (DLS) which was used to measure the particle size and size distribution of molecules or particles. The particle size information can be obtained by measuring the Brownian motion information of particles (Zetasizer Nano ZS90, Malvern,United Kingdom).

### MiRNAs isolation and quantitative real-time PCR

MiRNAs (miR-660-5p, miR-1258, miR-182-3p, miR-148a-3p, miR-27a-5p and miR-497-5p) in serum exosomes and serum were extracted by the miRNeasy Mini Kit (Qiagen, Toronto, Canada). 200 ng of total RNAs from each sample were reversely transcribed into complementary DNA (cDNA) using Mir-X miRNA First-Strand Synthesis Kit (TAKARA, Tokyo, Japan). Quantitative real-time PCR (qRT-PCR) was performed with SYBR Green PCR master mix (Applied Biosystems, California, America) on ABI 7500 fast real-time PCR system (Applied Biosystems, California, America) in a 20μL reaction system. U6 RNA was served as an endogenous control. 2^-△△CT^ method was used to calculate miRNA expression level in each sample. The stem ring specific primers and real time PCR primers were synthesized by Sangon Biotech (Sangon Biotech, Shanghai, China). The primer sequences are listed in Table 1.

**Table 1:** The Sequences of qRT-PCR Primers.

Gene symbol	Reverse transcription primer	PCR primer(5’-3’)
miR-660-5p	GTCGTATCCAGTGCAGGGTCCGAGGTATTCGCACTGGATACGACCAACTC	CGCGTACCCATTGCATATCG
miR-1258	GTCGTATCCAGTGCAGGGTCCGAGGTATTCGCACTGGATACGACTTCCAC	CGCGCGAGTTAGGATTAGGTC
miR-182-3p	GTCGTATCCAGTGCAGGGTCCGAGGTATTCGCACTGGATACGACTAGTTG	GCGCGTGGTTCTAGACTTGC
miR-148b-3p	GTCGTATCCAGTGCAGGGTCCGAGGTATTCGCACTGGATACGACACAAAG	CGCGTCAGTGCATCACAGAA
miR-27a-5p	GTCGTATCCAGTGCAGGGTCCGAGGTATTCGCACTGGATACGACTGCTCA	GCGAGGGCTTAGCTGCTTG
miR-497-5p	GTCGTATCCAGTGCAGGGTCCGAGGTATTCGCACTGGATACGACACAAAC	GCGCAGCAGCACACTGTG
U6	GTCGTATCCAGTGCAGGGTCCGAGGTATTCGCACTGGATACGACAAAATA	AGAGAAGATTAGCATGGCCCCTG

### Mathematical models establishment

Python (https:// scikit-learn.org/stable/) was used for all the mathematical models and decision tree establishment. The accuracy and the reliability of the models was verified by fivefold cross-validation. We developed 7 models to differentiate AECOPD from COPD using miR-1258 and routine laboratory tests: (1) support vector machine (SVM); (2) Random forest; (3) Gradient boosting classifier; (4) K nearest neighbors; (5) artificial neuron networks; (6) Gaussian Naïve Bayes; and (7) logistic regression model. We also built decision trees, Gini coefficient is a measure of data purity in a data set. The smaller the Gini coefficient is, the better the data purity is. When the Gini coefficient is 0, it indicates that the data of this set is 100% pure.

### Statistic analysis

Data was reported as mean ± SD and was analyzed using SPSS version 26.0 (IBM, New York, United States). The t-test and Man-Whitney U-test were used for the comparison of continuous variables while the chi-square test was used for the comparison of categorical variables between two groups. MedCalc 19.6.4 (MedCalc, Ostend, Belgium) was used to analyze receiver operating characteristic (ROC) which were applied to evaluate the sensitivity and specificity of diagnostic indicators. Logistic regression analysis was performed with language R (Microsoft, Washington D.C, America) to evaluate the correlation between miR-1258 and other indicators. *p* < 0.05 was considered statistically significant.

### Ethics approval

This study was performed in accordance with the Declaration of Helsinki and was approved by Peking University Third Hospital Medical Science Research Ethics Committee—approval: MR-11-22-001995.

## Results

### Characteristics of study population

42 patients with SCOPD, 66 patients with AECOPD and 45 normal controls (NC) were enrolled in our study. The basic demographic characteristics, laboratory results and comorbidities of the subjects were summarized in Table 2. Males in AECOPD groups were higher than those in SCOPD and NC. The levels of white blood cells (WBC), neutrophil count, neutrophil and lymphocyte ratio (NLR), CRP and PCT were significantly higher in patients with AECOPD than those in NC and patients with SCOPD (*p* < 0.05). AECOPD patients had higher rates of respiratory failure and cerebrovascular disease than SCOPD patients (*p* < 0.05).

**Table 2:** Basal characteristics of study population.

	NC (N = 45)	SCOPD (N = 42)	AECOPD (N = 66)	*p* value NC VS SCOPD	*p* value NC VS AECOPD	*p* value SCOPD VS AECOPD
Age (years)	75.9 ± 2.66	78.4 ± 10.11	77.1 ± 9.12	0.758	0.802	0.889
Sex n (%)						
Male	22 (48.9)	30 (71.4)	58 (87.9)	0.052	< 0.001	0.047
Female	23 (51.1)	12 (28.6)	8 (12.1)			
WBC (× 10^9^)	5.96 ± 1.31	6.49 ± 2.48	7.86 ± 3.52	0.215	< 0.001	0.010
NEU (× 10^9^/L)	3.53 ± 1.03	4.19 ± 1.68	6.34 ± 3.42	0.032	< 0.001	< 0.001
NLR	2.12 ± 0.386	3.29 ± 2.71	9.25 ± 13.39	0.009	< 0.001	< 0.001
EO (× 10^9^/L)	0.13 ± 0.103	0.222 ± 0.529	0.15 ± 0.175	0.259	0.535	0.295
CRP (mg/dl)	0.46 ± 0.35	1.17 ± 1.23	3.15 ± 4.34	< 0.001	< 0.001	< 0.001
PCT (μg/L)	0.068 ± 0.046	0.108 ± 0.139	0.20 ± 0.343	0.071	0.003	0.016
LDH (U/L)	153.6 ± 16.55	208.8 ± 67.5	196.9 ± 44.8	< 0.001	< 0.001	0.422

	SCOPD(%)	AECOPD(%)	*p* value			
Comorbidities and complications						
Respiratory failure	1 (0.03)	36 (0.61)	< 0.001			
Chronic pulmonary heart disease	0 (0)	9 (0.15)	0.05			
Autoimmune disease	1 (0.03)	2 (0.03)	1.00			
Cardiovascular disease	13 (0.41)	33 (0.56)	0.163			
Endocrine metabolic disease	4 (0.13)	17 (0.29)	0.078			
Cerebrovascular disease	1 (0.03)	14 (0.24)	0.011			
Neuropsychological disease	3 (0.09)	8 (0.14)	0.804			
Chronic nephrosis	2 (0.06)	8 (0.14)	0.476			
Chronic liver disease	0 (0)	4 (0.07)	0.332			
Chronic gastric disease	0 (0)	4 (0.07)	0.332			

*WBC* White blood cells, *NEU* Neutrophil, NLR Neutrophil to lymphocyte ratio, *EO* Eosinophil, *CRP *C-reaction protein, *PCT* Procalcitonin, *LDH* Lactate dehydrogenase.

### Characterization of serum exosomes

**Figure 1:**
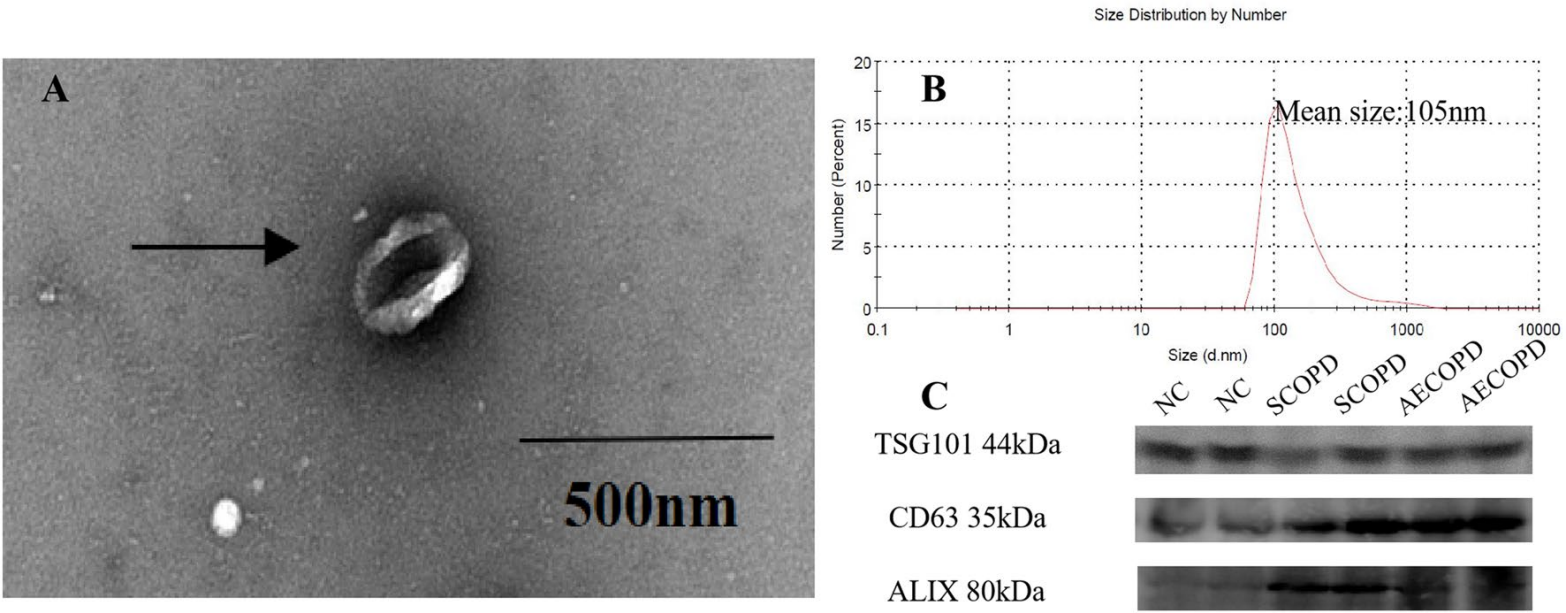
Identification of serum exosomes. (**A**) Serum exosomes were observed by transmission electron microscopy (TEM) (scale bar = 500 nm); (**B**) Size distribution of serum exosomes was detected by dynamic light scattering (DLS), and the size of serum exosomes was 105 ± 11.25 nm; (**C**) The expression of TSG101, CD63 and ALIX in serum exosomes from three groups were detected by western blot.

Serum exosomes isolated and extracted from serum were identified by TEM, western blot and Zeta sizer Nano tracking analysis. As shown in Fig. 1, serum exosomes were a kind of vesicles with bilayer membrane, had a mean diameter of 105 ± 11.25 nm and expressed specific exosomes’ proteins (CD63, TSG101, ALIX). According to the particle size, morphology and expression of protein markers, it could be confirmed that exosomes from the serum were isolated successfully. We compared the size distribution for all measured groups (NC, SCOPD, AECOPD) and found no difference among the groups (*p* > 0.05).

### Expression of miRNAs

**Figure 2:**
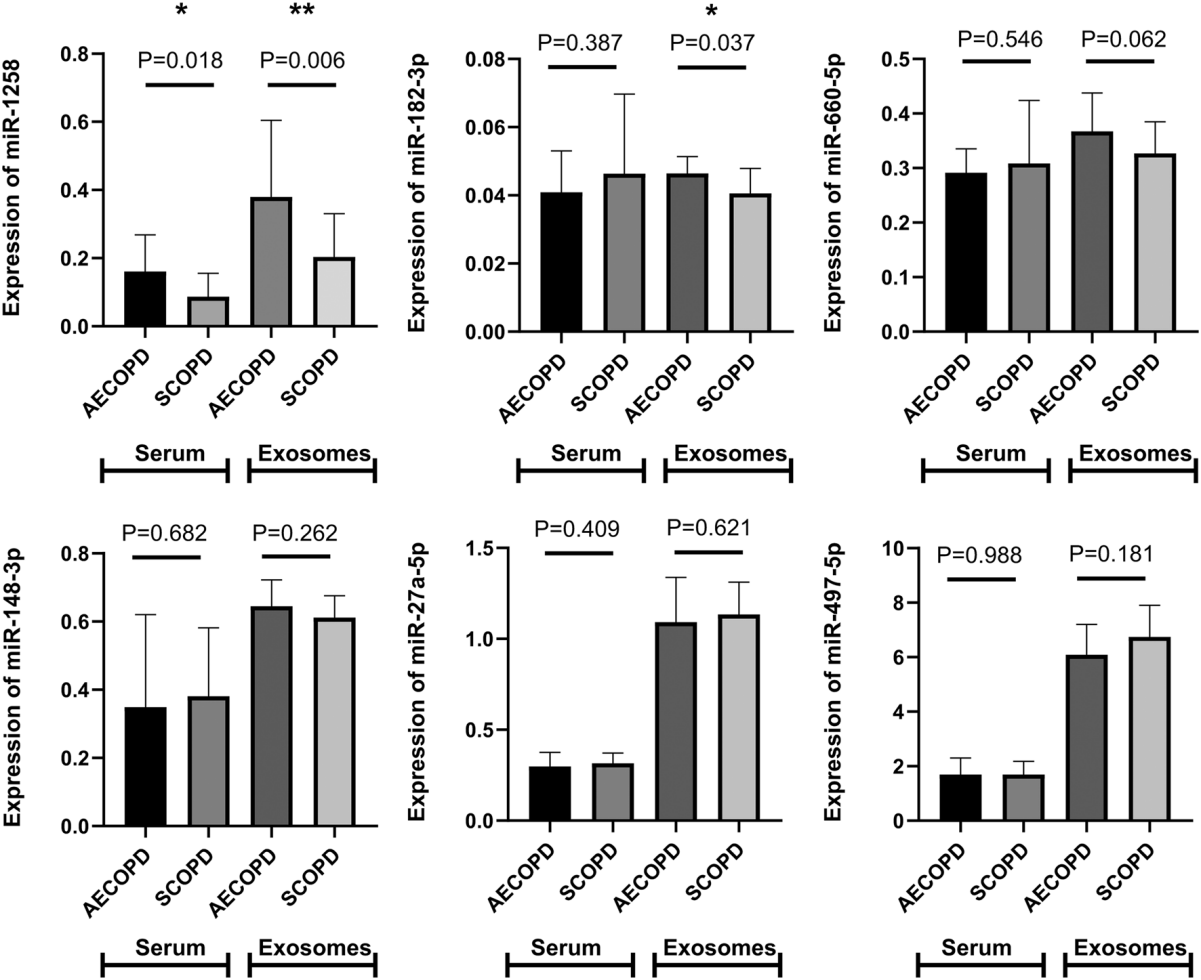
The expression of miRNAs (miR-1258, miR-182-3p, miR-148a-3p, miR-27a-5p,miR-660-5p and miR-497-5p) in SCOPD (n = 18) and AECOPD (n = 19) patients in pre-experiment. *: the difference between different groups was statistically significant (*p* < 0.05); **: the difference between different groups was statistically significant (*p* < 0.01).

**Figure 3:**
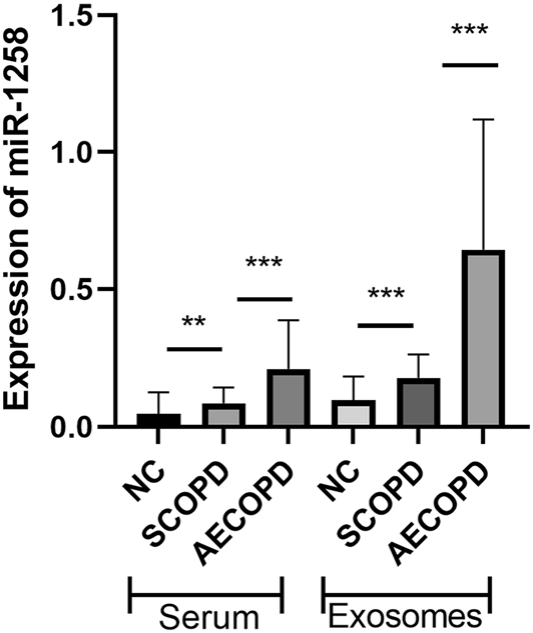
The expression of miR-1258 in normal controls (n = 45), SCOPD (n = 42) and AECOPD patients (n = 66). **: the difference between different groups was statistically significant (*p* < 0.01); ***: the difference between different groups was statistically significant (*p* < 0.001).

To screen the probable biomarkers of AECOPD, the expression of miRNAs (miR-660-5p, miR-1258, miR-182-3p, miR-148a-3p, miR-27a-5p and miR-497-5p) in serum and serum exosomes from SCOPD (n = 18) and AECOPD patients (n = 19) were detected by qRT-PCR. It was found that expression of miR-1258 was significantly higher in both serum and serum exosomes of AECOPD patients than that of SCOPD patients (serum: 0.161 ± 0.108 vs 0.087 ± 0.069; exosome: 0.380 ± 0.225 vs 0.204 ± 0.126, *p* < 0.001), and there have no significant difference in other molecules.(Fig. 2,Table 3). Based on these findings, we further detected all the samples from AECOPD (n = 66), SCOPD patients (n = 42) and NCs (n = 45), and found that the expression of miR-1258 in both serum and serum exosomes was the highest in patients with AECOPD (0.21 ± 0.176 in serum, 0.386 ± 0.285 in serum exosomes), followed by the patients with SCOPD (0.086 ± 0.057 in serum, 0.264 ± 0.13 in serum exosomes), and the level in NC was the lowest (0.047 ± 0.078 in serum, 0.146 ± 0.386 in serum exosomes), the differences among the three groups were statistically significant (*p* < 0.001) (Fig. 3).

**Table 3:** Pre-experienment for choosing biomarker to diagnose SCOPD from AECOPD.

	Serum			Exosome		
	AECOPD(n = 19)	SCOPD(n = 18)	*p* value	AECOPD(n = 19)	SCOPD(n = 18)	*p* value
miR-1258	0.161 ± 0.108	0.087 ± 0.069	0.018	0.380 ± 0.225	0.204 ± 0.126	0.006
miR-182-3p	0.041 ± 0.012	0.046 ± 0.023	0.387	0.047 ± 0.005	0.041 ± 0.007	0.037
miR-660-5p	0.291 ± 0.044	0.309 ± 0.115	0.546	0.367 ± 0.070	0.327 ± 0.058	0.062
miR-148-3p	0.349 ± 0.271	0.381 ± 0.200	0.682	0.645 ± 0.077	0.612 ± 0.064	0.262
miR-27a-5p	0.298 ± 0.078	0.316 ± 0.055	0.409	1.091 ± 0.247	1.135 ± 0.177	0.621
miR-497-5p	0.696 ± 0.610	1.698 ± 0.482	0.988	6.089 ± 1.120	6.742 ± 1.160	0.181

### The diagnostic value of various biomarkers for AECOPD

**Figure 4:**
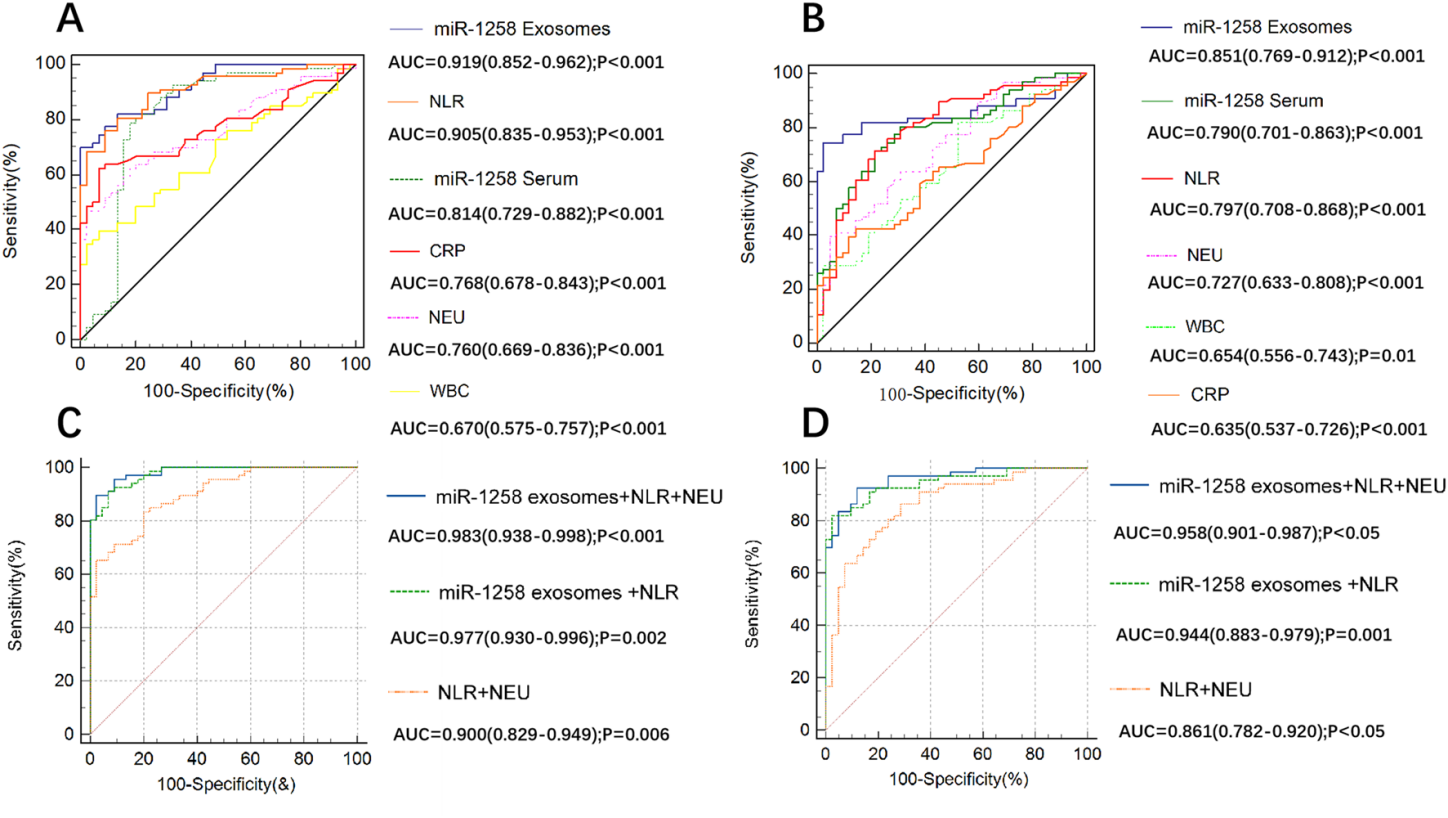
Receiver operating characteristic (ROC) curve analysis of serum and exosomal miR-1258 and other biomarkers to diagnose AECOPD. (**A**) Discriminate AECOPD from normal controls; (**B**) discriminate AECOPD from SCOPD; (C) the combined detection of exosomal miR-1258 and NLR to discriminate AECOPD from normal controls; (**D**) the combined detection of exosomal miR-1258 and NLR to discriminate AECOPD from SCOPD. Abbreviation: AUC: area under the curve; NLR: neutrophil to lymphocyte ratio; CRP: C-reactive protein; WBC; white blood cell count; NEU: neutrophil count.

To evaluate the performance of miR-1258 in diagnosis AECOPD, ROC was drew, area under curve (AUC) and cut off value were calculated and compared. Serum exosomes miR-1258 was demonstrated to be the most effective for differential diagnosis AECOPD patients from normal people and SCOPD patients, the AUC was 0.919 in AECOPD and NC, the AUC was 0.851 in AECOPD and SCOPD. The optimal cut-off values, specificity and sensitivity of various biomarkers were shown in Table 4 and Fig. 4A, B. Among them, exosomal miR-1258 showed the highest specificity in differentiating AECOPD from SCOPD (97.62%) and NC (100%), and with a higher sensitivity of 74.2% in identifying AECOPD from SCOPD.

**Table 4:** The performance of various biomarkers in differentiating AECOPD from normal controls (NC) and SCOPD.

	AECOPD vs NC					AECOPD vs SCOPD				
	AUC	95% CI	Sensitivity	Specificity	Cut off	AUC	95% CI	Sensitivity	Specificity	Cut off
Exosome miR-1258 + NLR + NEU	0.983	0.938–0.998	89.39	97.78	∕	0.958	0.901–0.987	92.42	88.10	∕
Exosome miR-1258 + NLR	0.977	0.930–0.996	90.91	93.3	∕	0.944	0.883–0.979	81.8	97.6	∕
NLR + NEU	0.900	0.829–0.949	83.33	80.00	∕	0.861	0.782–0.920	86.36	71.43	∕
Exosome miR-1258	0.919	0.852–0.962	76.8	100	0.163	0.851	0.769–0.912	74.2	97.6	0.193
NLR	0.905	0.835–0.953	80.3	86.7	3.03	0.797	0.708–0.868	71.2	78.6	3.57
Serum miR-1258	0.814	0.729–0.882	97.0	84.4	0.045	0.790	0.701–0.863	71.2	78.57	0.117
CRP	0.768	0.678–0.843	62.1	93.3	0.83	0.635	0.537–0.726	42.4	85.8	2.26
NEU	0.760	0.669–0.836	56.1	97.8	5.14	0.727	0.633–0.808	39.4	95.2	6.38
WBC	0.670	0.575–0.757	39.4	97.8	8.49	0.654	0.556–0.743	81.8	47.6	5.5

#### The diagnostic efficacy of exosomal miR-1258 combined with other conventional indicators

When analyzing the diagnostic value of various biomarkers through AUC, it was found that exosomal miR-1258 and NLR were the most excellent and significantly superior to other indicators. To improve the diagnostic efficacy, we further performed ROC curve analysis on the combination of these two indicators (Fig. 4C, D). We did find that exosomal miR-1258 combined with NLR increased the AUC to 0.977 (AECOPD vs NC) and 0.944 (AECOPD vs SCOPD). Diagnositic efficiency of the combination of NLR + NEU (AECOPD vs NC 0.900, AECOPD vs SCOPD 0.861) and exosomal miR-1258 + NLR + NEU (AECOPD vs NC AUC 0.983, AECOPD vs SCOPD 0.958), as shown in Fig. 4, could prove that the exosomal miR-1258 + NLR + NEU was superior to that of NLR + NEU, and there was a statistically significant difference (*p* < 0.01). The combination diagnostic efficiency has been improved significantly after adding the biomarker exosomal miR-1258, which demonstrated that exosomal miR-1258 is an effective biomarker for predicting AECOPD.

### The diagnostic models of AECOPD

We further used scikit learn, a package of python, to select the model with the best accuracy. Based on foregoing results, exosomal miR-1258 and NLR were the most excellent, significantly superior to other biomarkers. We also added other indicators (NEU, CRP) with these two best indicators and analyzed different combinations in order to choose the best model. We adopted the method of fivefold cross-validation to evaluate the validity of the model, and found that the logistics regression model showed the best accuracy in identifying AECOPD and SCOPD, among which miR1258 + NLR + NEU and miR1258 + NLR + NEU + CRP obtained the highest accuracy of 0.880 ± 0.061 (as shown in Table 5). Therefore, based on the results of logistics regression, SCOPD was used as the control group, we established a formula to predict the probability of AECOPD:

$$P = \frac{{\exp \left( {2.35*\frac{MiR1258 - 0.23}{{0.22}} + 0.82*\frac{NEU - 5.50}{{3.04}} + 0.42*\frac{CRP - 2.38}{{3.58}} + 2.06*\frac{NLR - 6.93}{{10.92}} + 1.63} \right)}}{{\exp \left( {2.35*\frac{MiR1258 - 0.23}{{0.22}} + 0.82*\frac{NEU - 5.50}{{3.04}} + 0.42*\frac{CRP - 2.38}{{3.58}} + 2.06*\frac{NLR - 6.93}{{10.92}} + 1.63} \right) + 1}}$$

**Table 5:** fivefold cross-validation of models for AECOPD diagnosis.

	Cross-validation(± SD)	
	NC and AECOPD	SCOPD and AECOPD
Support Vector Machine		
Exosomal MiR-1258	0.794 ± 0.079	0.824 ± 0.053
NLR	0.774 ± 0.065	0.703 ± 0.111
MiR-1258 + NLR	0.883 ± 0.046	0.833 ± 0.085
MiR-1258 + NLR + NEU	0.937 ± 0.047	0.861 ± 0.049
MiR-1258 + NLR + NEU + CRP	0.919 ± 0.053	0.852 ± 0.053
NLR + NEU + CRP	0.828 ± 0.073	0.723 ± 0.086
Random forest		
Exosomal MiR-1258	0.821 ± 0.046	0.852 ± 0.036
NLR	0.802 ± 0.059	0.595 ± 0.118
MiR-1258 + NLR	0.937 ± 0.021	0.852 ± 0.108
MiR-1258 + NLR + NEU	0.928 ± 0.036	0.805 ± 0.062
MiR-1258 + NLR + NEU + CRP	0.919 ± 0.018	0.842 ± 0.095
NLR + NEU + CRP	0.864 ± 0.042	0.629 ± 0.091
Gradient boosting classifier		
Exosomal MiR-1258	0.776 ± 0.093	0.824 ± 0.053
NLR	0.775 ± 0.080	0.722 ± 0.143
MiR-1258 + NLR	0.919 ± 0.032	0.814 ± 0.115
MiR-1258 + NLR + NEU	0.937 ± 0.036	0.842 ± 0.070
MiR-1258 + NLR + NEU + CRP	0.919 ± 0.044	0.806 ± 0.060
NLR + NEU + CRP	0.847 ± 0.043	0.731 ± 0.058
Logistic regression		
Exosomal MiR-1258	0.839 ± 0.070	0.806 ± 0.050
NLR	0.784 ± 0.071	0.731 ± 0.091
MiR-1258 + NLR	0.910 ± 0.029	0.843 ± 0.098
MiR-1258 + NLR + NEU	0.911 ± 0.062	0.880 ± 0.061
MiR-1258 + NLR + NEU + CRP	0.919 ± 0.043	0.880 ± 0.061
NLR + NEU + CRP	0.829 ± 0.071	0.758 ± 0.095
K nearest neighbors		
Exosomal MiR-1258	0.777 ± 0.107	0.797 ± 0.046
NLR	0.730 ± 0.029	0.658 ± 0.136
MiR-1258 + NLR	0.874 ± 0.051	0.825 ± 0.110
MiR-1258 + NLR + NEU	0.910 ± 0.039	0.852 ± 0.089
MiR-1258 + NLR + NEU + CRP	0.910 ± 0.057	0.861 ± 0.083
NLR + NEU + CRP	0.865 ± 0.056	0.731 ± 0.081
Artificial neuron networks		
Exosomal MiR-1258	0.775 ± 0.085	0.824 ± 0.053
NLR	0.784 ± 0.087	0.722 ± 0.143
MiR-1258 + NLR	0.928 ± 0.037	0.814 ± 0.115
MiR-1258 + NLR + NEU	0.928 ± 0.034	0.842 ± 0.070
MiR-1258 + NLR + NEU + CRP	0.910 ± 0.029	0.806 ± 0.060
NLR + NEU + CRP	0.856 ± 0.019	0.731 ± 0.058
Gaussian Naïve Bayes		
Exosomal MiR-1258	0.776 ± 0.093	0.816 ± 0.038
NLR	0.775 ± 0.080	0.648 ± 0.099
MiR-1258 + NLR	0.919 ± 0.032	0.815 ± 0.078
MiR-1258 + NLR + NEU	0.937 ± 0.036	0.833 ± 0.075
MiR-1258 + NLR + NEU + CRP	0.919 ± 0.044	0.787 ± 0.057
NLR + NEU + CRP	0.847 ± 0.043	0.667 ± 0.072

In multivariate logistic regression analysis, exosomal and serum miR-1258, NLR, NEU, CRP, WBC, PCT, sex, Respiratory failure and Cerebrovascular Disease were identified as independent predictors for AECOPD (Table 6). Among all the indicators, exosomal miR-1258 showed the highest odds ratio, indicating a strong association between high exosomal miR-1258 and AECOPD.

**Table 6:** Logistic regression analysis of variables in predicting the AECOPD.

Variables	OR (95%CI)	*p* value
Exosomal miR-1258	9.8 × 10^9^(4.579 × 10^3^–2.09 × 10^14^)	< 0.001
Serum miR-1258	2.39 × 10^12^(2.32 × 10^4^–2.46 × 10^20^)	< 0.01
NLR	1.738(1.223–2.472)	< 0.01
NEU	2.537(0.619–10.398)	0.196
CRP	1.427 (0.79–2.579)	0.239
WBC	0.607(0.188–1.963)	0.404
PCT	0.822(0.148–4.555)	0.822
Sex	3.371(1.203–9.449)	0.021
Respiratory failure	41.868(5.321–329.445)	< 0.001
Cerebrovascular Disease	1.806(0.652–5.004)	0.256

**Figure 5:**
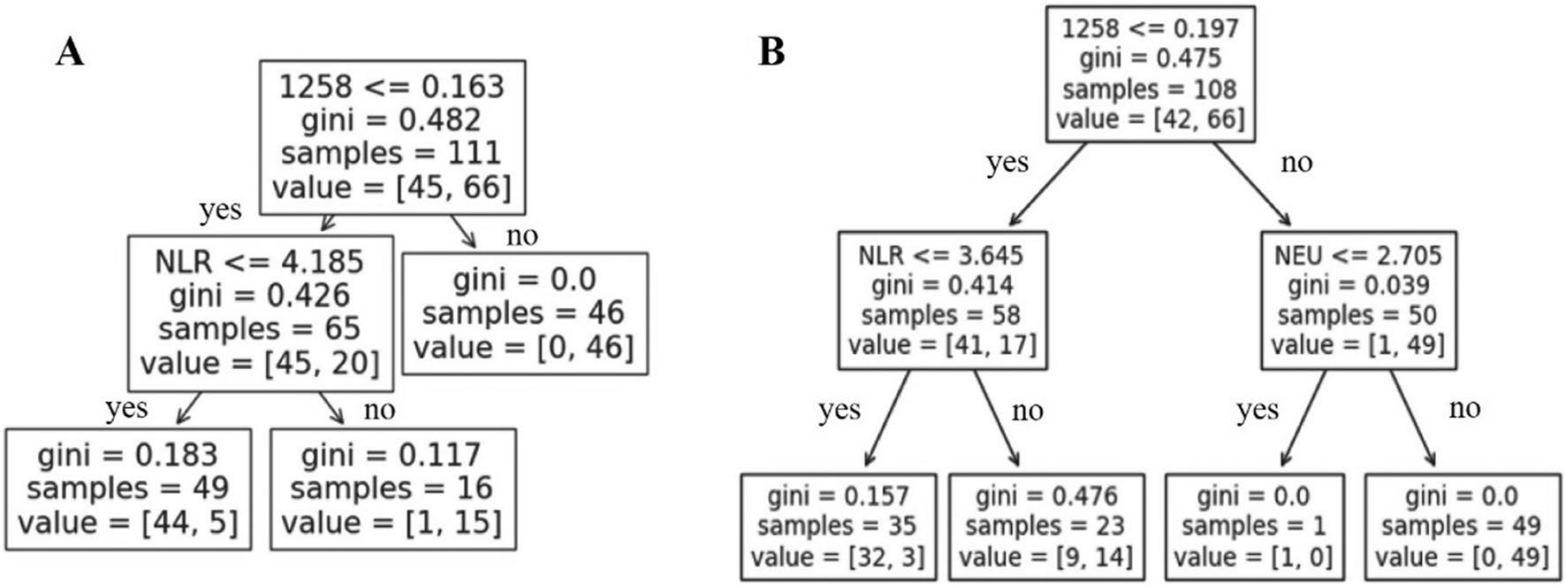
Decision tree to identify AECOPD based on the test results of exosomal miR-1258, NLR and / or neutrophil count. (**A**) Identify AECOPD from normal controls; (**B**) Identify AECOPD from SCOPD. Abbreviation: 1258: exosomal miR-1258; gini: gini coefficient; samples: the number of samples that meet the criteria. value = [the number of normal controls (**A**) or patients with SCOPD (**B**), the number of patients with AECOPD.

Decision tree is helpful to establish clinical diagnosis strategy. According to the indexes selected and verified by logistic regression model mentioned above, decision trees were built up. We found that of the 108 patients with SCOPD and AECOPD, 50 patients with miR-1258 greater than 0.197, 49 (98%) of them had a neutrophil count greater than 2.705 × 10^9^/L were diagnosed with AECOPD in the training set, only one patient was diagnosed with SCOPD (gini coefficient was 0.0). While among the 58 patients with exosomal miR-1258 ≤ 0.197, 35 patients had NLR ≤ 3.645, 32 (91.4%) of whom were diagnosed as SCOPD, and only 3 patients developed acute exacerbation (gini coefficient was 0.157) (Fig. 5). It further confirmed the important role of exosomal miR-1258, NLR and neutrophil count in the diagnosis of AECOPD.

### MiR-1258 expression in different severities and phenotypes of AECOPD

To evaluate the association between miR-1258 and AECOPD severity, phenotypes (e.g., eosinophilic and frequent exacerbations), and radiographic features (e.g., emphysema), the levels of serum and exosomal miR-1258 were compared between AECOPD patients with and without respiratory failure, ICU admission, emphysema, and exacerbations in the past year, however, no statistical difference was found (Table 7).

**Table 7:** The expression of miR-1258 in different severities and phenotypes of AECOPD.

	Serum miR-1258		*p* value	Exosomal miR-1258		*p* value
	With	Without		With	Without	
Exacerbations in the past year	0.237 ± 0.209	0.197 ± 0.163	0.421	0.286 ± 0.186	0.353 ± 0.264	0.319
Respiratory failure	0.185 ± 0.149	0.213 ± 0.191	0.393	0.411 ± 0.282	0.365 ± 0.265	0.454
ICU admission	0.203 ± 0.181	0.214 ± 0.176	0.811	0.333 ± 0.253	0.315 ± 0.231	0.766
Emphysema	0.186 ± 0.144	0.30 ± 0.268	0.178	0.347 ± 0.256	0.256 ± 0.173	0.253
Eosinophil counts	0.182 ± 0.100	0.211 ± 0.090	0.482	0.432 ± 0.226	0.478 ± 0.318	0.668

### Correlations of exosomal miR-1258 and other biomarkers

**Figure 6:**
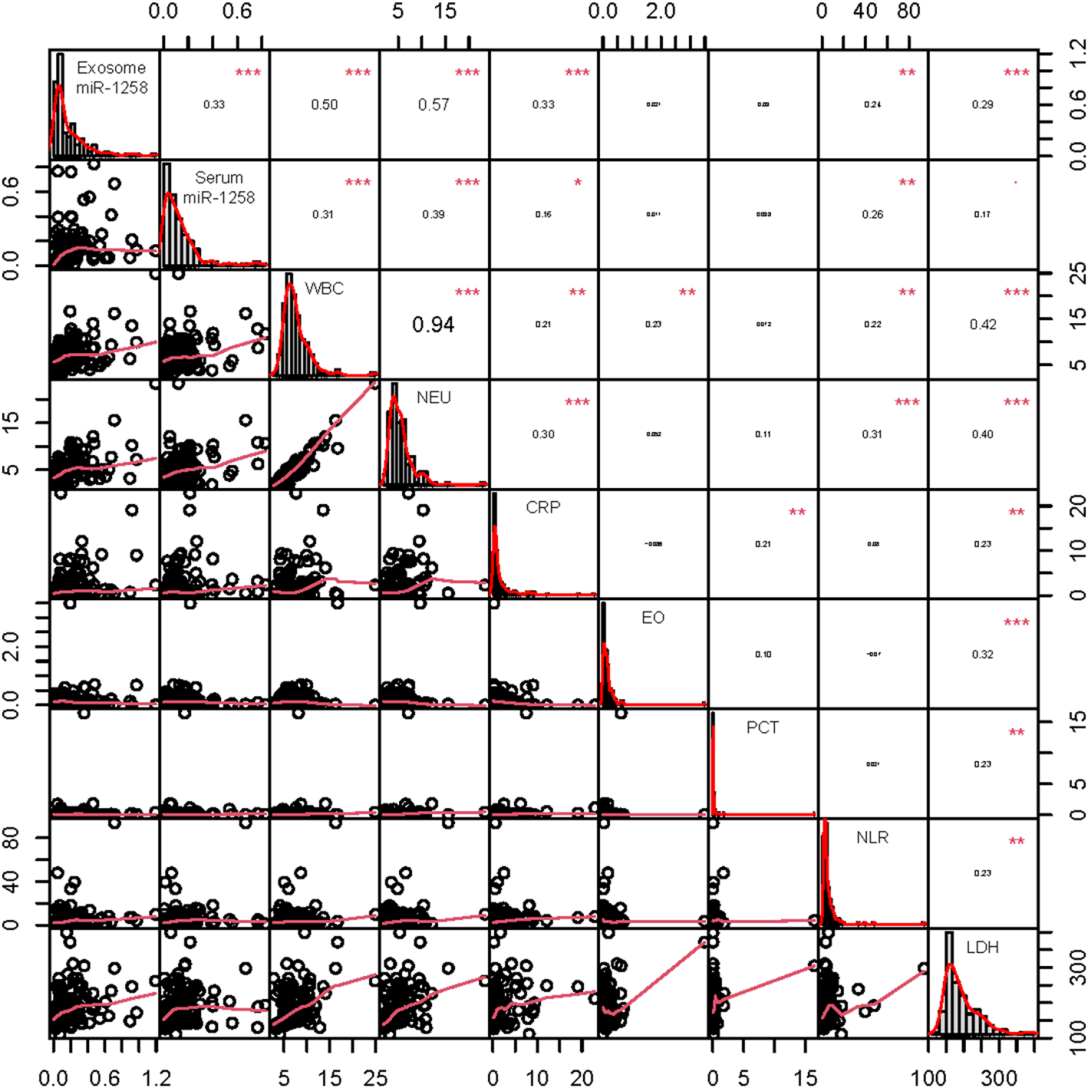
Correlation analysis of different biomarkers in the study population. Serum exosomes miR-1258 was positively correlated with serum miR-1258(r = 0.33, *p* < 0.001), neutrophil count (r = 0.57, *p* < 0.001), WBC (r = 0.50, *p* < 0.001), CRP (r = 0.33, *p* < 0.001), lactic dehydrogenase (LDH) (r = 0.29, *p* < 0.001), and NLR (r = 0.24, *p* < 0.05). **: the difference between different groups was statistically significant (*p* < 0.01); ***: the difference between different groups was statistically significant (*p* < 0.001).

Correlations of various biomarkers were analyzed by language R and the results were shown in Fig. 6. Exosomal miR-1258 was positively correlated with serum miR-1258 (r = 0.33, *p* < 0.001) neutrophil count (r = 0.57, *p* < 0.001), WBC (r = 0.50, *p* < 0.001), CRP (r = 0.33, *p* < 0.001), LDH (r = 0.29, *p* < 0.001) and NLR (r = 0.24, *p* < 0.001). No significant correlations were found between exosomal miR-1258 and eosinophil count (r = 0.021, *p* > 0.05) and PCT (r = 0.09, *p* > 0.05). Meanwhile, correlation index between exosomal miR-1258 and other indicators were higher than serum. Correlation index and *p* value between exosomal miR-1258 and other indicators were shown in Table 8.

**Table 8:** Correlation index between exosomal miR-1258 and other indicators.

r^2^/*p* value	Exosome miR-1258	Serum miR-1258	WBC	NEU	CRP	EO	PCT	NLR	LDH
Exosome miR-1258	∕	0.33 < 0.001	0.51 *p* < 0.001	0.57 *p* < 0.001	0.33 *p* < 0.001	0.02 0.80	0.09 0.27	0.24 *p* < 0.001	0.29 *p* < 0.001
Serum miR-1258	0.33 *p* < 0.001	∕	0.31 *p* < 0.001	0.39 *p* < 0.001	0.16 *p* < 0.05	0.01 0.89	0.04 0.24	0.26 *p* < 0.01	0.17 0.054
WBC	0.50 *p* < 0.001	0.31 *p* < 0.001	∕	0.94 *p* < 0.001	0.21 *p* < 0.01	0.23 *p* < 0.01	0.07 0.3	0.22 *p* < 0.01	0.42 *p* < 0.001
NEU	0.57 *p* < 0.001	0.39 *p* < 0.001	0.94 *p* < 0.001	∕	0.30 *p* < 0.001	0.05 0.52	0.11 0.16	0.31 *p* < 0.001	0.40 *p* < 0.001
CRP	0.33 *p* < 0.001	0.16 *p* < 0.05	0.21 *p* < 0.01	0.30 *p* < 0.001	∕	-0.09 0.29	0.21 *p* < 0.01	0.08 0.32	0.23 *p* < 0.01
EO	0.02 0.80	0.01 0.89	0.23 *p* < 0.01	0.05 0.52	-0.09 0.29	∕	0.10 0.20	-0.07 0.39	0.31 *p* < 0.001
PCT	0.10 0.26	0.04 0.64	0.07 0.37	0.11 0.16	0.21 *p* < 0.01	0.10 0.20	∕	0.02 0.79	0.23 *p* < 0.01
NLR	0.24 *p* < 0.01	0.26 *p* < 0.01	0.22 *p* < 0.01	0.31 *p* < 0.001	0.08 0.33	-0.07 0.39	0.02 0.79	∕	0.23 *p* < 0.01
LDH	0.29 *p* < 0.001	0.17 0.054	0.42 *p* < 0.001	0.40 *p* < 0.001	0.23 *p* < 0.01	0.32 *p* < 0.001	0.23 *p* < 0.01	0.23 *p* < 0.01	∕

*WBC* White blood cells, *NEU* Neutrophil, *CRP* C-reaction protein, *EO* Eosinophil, *PCT* Procalcitonin, *NLR* Neutrophil to lymphocyte ratio, *LDH* Lactate dehydrogenase.

## Discussion

Prior reports have shown that miRNAs play an essential role in regulating gene expression in several different physiological and pathophysiological conditions, including chronic inflammatory lung diseases, such as SCOPD^20–25^. However, the role of miRNA, especially exosomal miRNAs, in the diagnosis, severity assessment and phenotype differentiation of AECOPD has been rarely reported^27,28^. In this study, we showed for the first time that miR-1258 could be a valuable biomarker in the AECOPD diagnosis (Supplementary Figures).

We found that miR-1258 in exosomal and serum from AECOPD patients (0.21 ± 0.176 in serum, 0.386 ± 0.285 in serum exosomes) were significantly higher than that from SCOPD (0.086 ± 0.057 in serum, 0.264 ± 0.13 in serum exosomes) and NC (0.047 ± 0.078 in serum, 0.146 ± 0.386 in serum exosomes) (Fig. 3). The AUC for exosomal miR-1258 was 0.851(95% CI 0.769–0.902, *p* < 0.001) and the cut-off value was 0.19 with 74.24% sensitivity and 97.62% specificity in discriminating AECOPD from SCOPD, which was better than the AUC for serum miR-1258 (AUC 0.790 (95% CI 0.701–0.863, *p* < 0.001), cut-off value 0.11 with 71.21% sensitivity and 78.57% specificity). Both exosomal and serum miR-1258 were better than other commonly used clinical indicators, including CRP, PCT, WBC, NLR. These results suggested that miR-1258 had the potential to serve as a biomarker to identify AECOPD. Compared with serum, the determination of exosomal miR-1258 was more accurate and reliable. serum exosomes refer to small membrane vesicles (30–150 nm) and are rich in proteins, DNA, mRNA, miRNA, circular RNA, etc., which can be easily degraded if directly exposed to blood and body fluids. serum exosomes composed of phospholipid bilayer provide a safe carrier. These substances can be taken up and absorbed by target cells as mediators, such as macrophages, renal tubular epithelial cells and endothelial cells, thus regulating the body’s injury process^29,30^.

Moreover, when combined exosomal miR-1258 and NLR, the AUC for discriminating AECOPD from SCOPD increase to 0.944, with sensitivity 81.82% and specificity 97.62%, which was superior to exosomal miR-1258 or NLR tested alone.

To verify the diagnostic value of miR-1258, seven mathematical models were established and the accuracy and reliability were evaluated. The results confirmed that exosomal miR-1258 was superior to NLR alone no matter which modes was performed, and the logistics regression model based on exosomal miR-1258, NLR and neutrophils count had the best predictive power for identifying AECOPD. The further constructed decision trees showed that based on the population in this study, according to miR-1258, NLR and neutrophil count, 49 (74.2%) out of 66 AECOPD were identified from SCOPD (gini coefficient = 0), which suggested exosomal miR-1258 could serve as a useful screening biomarker for AECOPD diagnosis and decision tree could provide a valuable reference for the formulation of clinical laboratory diagnostic strategies. To explore the possible origin or biological significance of miR-1258, we analyzed the relationships between miR-1258 and other indicators of AECOPD, and found that the level of exosomal miR-1258 was positively correlated with WBC counts, neutrophil counts, NLR, CRP and LDH in the total subjects. Whereas, in AECOPD patients, the positively correlations were only found between miR-1258 and WBC counts and neutrophil counts. Some research showed that serum exosomes and miRNAs inside were different in different diseases or healthy conditions, and the differentially expressed miRNAs could be due to different cell types (T lymphocytes, B lymphocytes and monocytes) present in the blood^30,31^ or bronchial epithelial cells^30^. Based on limited evidence, we could speculate that exosomal miR-1258 may be derived from peripheral blood WBCs; and reference to previous investigations on the causes of AECOPD and combined with the results of this study, it was reasonable to speculate that exosomal miR-1258 may be related to infection-induced systemic inflammation, while it still need further research to confirm.

However, no associations of miR-1258 with respiratory failure, ICU stay, exacerbations in previous year, blood eosinophil count and emphysema were found, which suggested that it seemed to be not appropriate to serve miRNA-1258 as a cue of AECOPD severity and clinical phenotype.

There were some limitations in our study. Firstly, it was single-center observational study with relatively small sample size, the clinical value of miR-1258 needs to be further confirmed in a larger prospective cohort study. Secondly, we screened multiple miRNAs in the pre-experiment, so the sample size of each group was relatively small. In the follow-up experiment, we only verified the miRNAs with the most significant changes with the samples of all enrolled patients, it might be better if all miRNAs were tested with all samples, and the results would be more convincing. Finally, the AECOPD patients enrolled were all hospitalized with severe exacerbations, therefore, this conclusion about exosomal miRNA-1258 may not be extended to the mild to moderate AECOPD patients; third, research related to the source of serum exosomes were not involved, so the possible underlying mechanisms could not be clearly elucidated. The studies in future should focus on these issues.

In conclusion, we confirmed that miR-1258 was significantly increased in AECOPD by experimental methods and mathematical models. It had the efficacy of diagnosing AECOPD, and serum exosomes were better than serum. When combined with NLR, its diagnostic performance was further improved. Therefore, miR-1258 may be a reliable and effective biomarker for the distinguishment of AECOPD. In addition, based on miR-1258, combined with other commonly used clinical indicators, we established a decision-making process which may be able to further improve the accuracy of AECOPD diagnosis.

### Supplementary Information

Supplementary Figures.

## Data Availability

The original contributions presented in the study are included in the article/Supplementary Material, further inquiries can be directed to the corresponding authors. The data and materials that support the findings of this study are available from the corresponding author upon reasonable request.

## Abbreviations

AECOPD: Acute exacerbations of chronic obstructive pulmonary disease
SCOPD: Stable chronic obstructive pulmonary disease
NC: Normal controls
PCR: Polymerase chain reaction
miRNAs: Micro RNAs
NLR: Neutrophil to lymphocyte ratio
NEU: Neutrophil
WBC: White blood cell
CRP: C-reactive protein
WHO: World Health Organization
PCT: Procalcitonin
UTR: Untranslated region
GOLD: Global Initiative for Chronic Obstructive Lung Disease
FEV: Forced expiratory volume
FVC: Forced vital capacity
HRCT: High-resolution computed tomography
PBS: Phosphate-buffered saline
TEM: Transmission electron microscopy
WB: Western blot
qRT-PCR: Quantitative real-time PCR
SVM: Support vector machine
ROC: Receiver operating characteristic
