# Supplementary material for: Serum exosomal microRNA-1258 may as a novel biomarker for the diagnosis of acute exacerbations of chronic obstructive pulmonary disease
Source: Sci Rep. 2023 Oct 26;13:18332. doi: 10.1038/s41598-023-45592-4 (PMC10603088; doi:10.1038/s41598-023-45592-4)
Supplement: Supplementary file 1 — Supplementary Figures. [file 41598_2023_45592_MOESM1_ESM.docx]

**S1. Statistic difference of different biomarkers in males and females.**

|  | Male(n=22) | Female(n=23) |  |
| --- | --- | --- | --- |
|  | X±SD | | P value |
| Exosome miR-1258 | 0.0509±0.009 | 0.0466±0.010 | 0.745 |
| Serum miR-1258 | 0.0541±0.073 | 0.0406±0.083 | 0.569 |
| WBC | 5.93±1.31 | 5.98±1.35 | 0.901 |
| NEU | 3.56±1.04 | 3.49±1.04 | 0.822 |
| CRP | 0.431±0.330 | 0.494±0.371 | 0.545 |
| EO | 0.146±0.112 | 0.116±0.095 | 0.325 |
| PCT | 0.065±0.039 | 0.070±0.052 | 0.741 |
| NLR | 2.21±1.03 | 1.97±0.941 | 0.439 |
| LDH | 152.91±16.01 | 154.26±17.38 | 0.788 |

**S2.** Serum exosomes were observed by transmission electron microscopy (TEM)

**
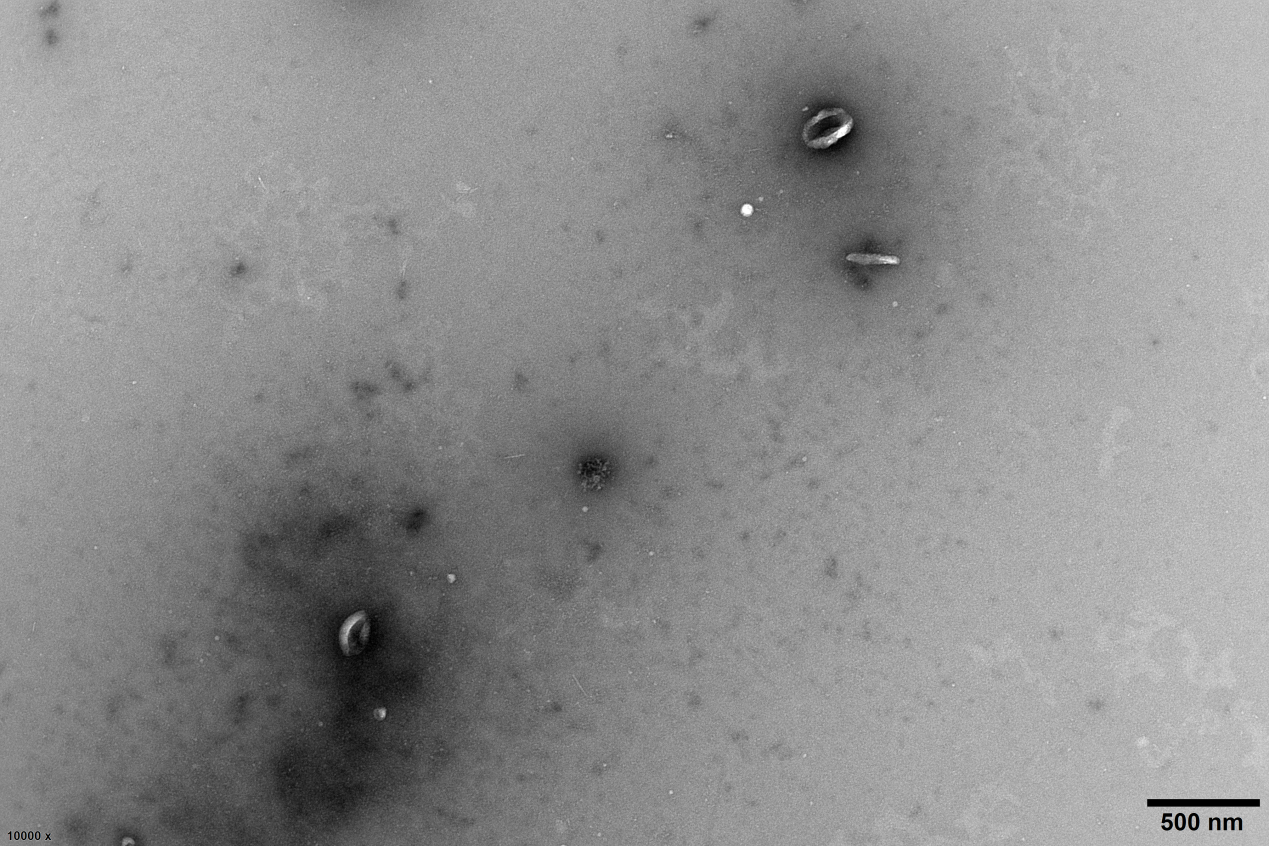
**

**S3. Original and unprocessed images of specific biomarkers in serum exosomes**

The earliest image


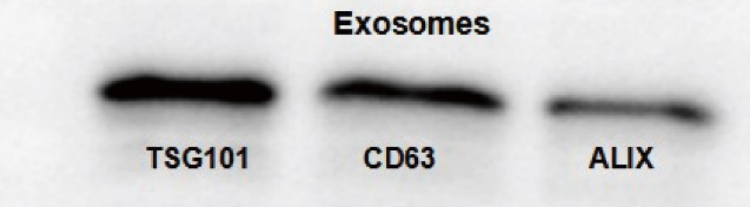


Three fully gels and blots images in last time revision


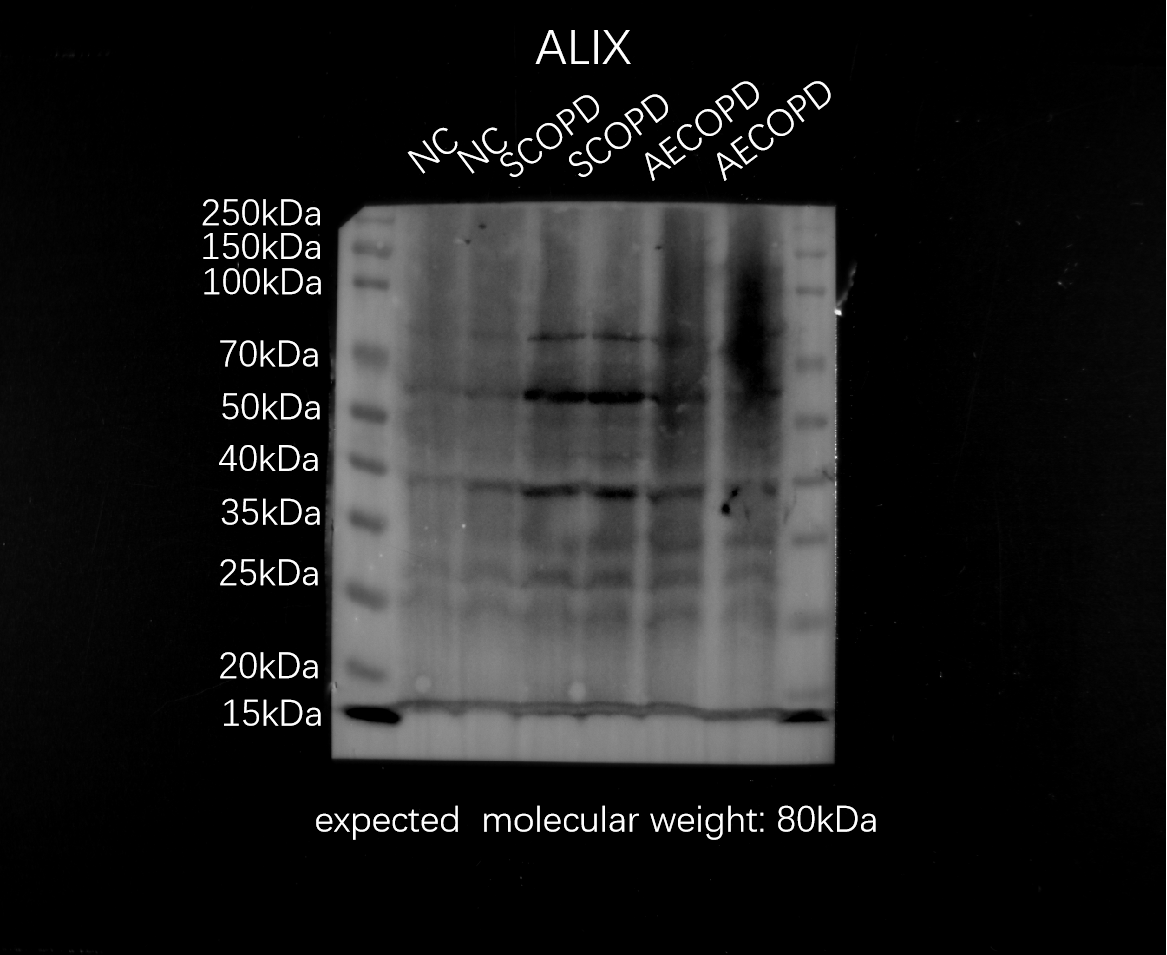

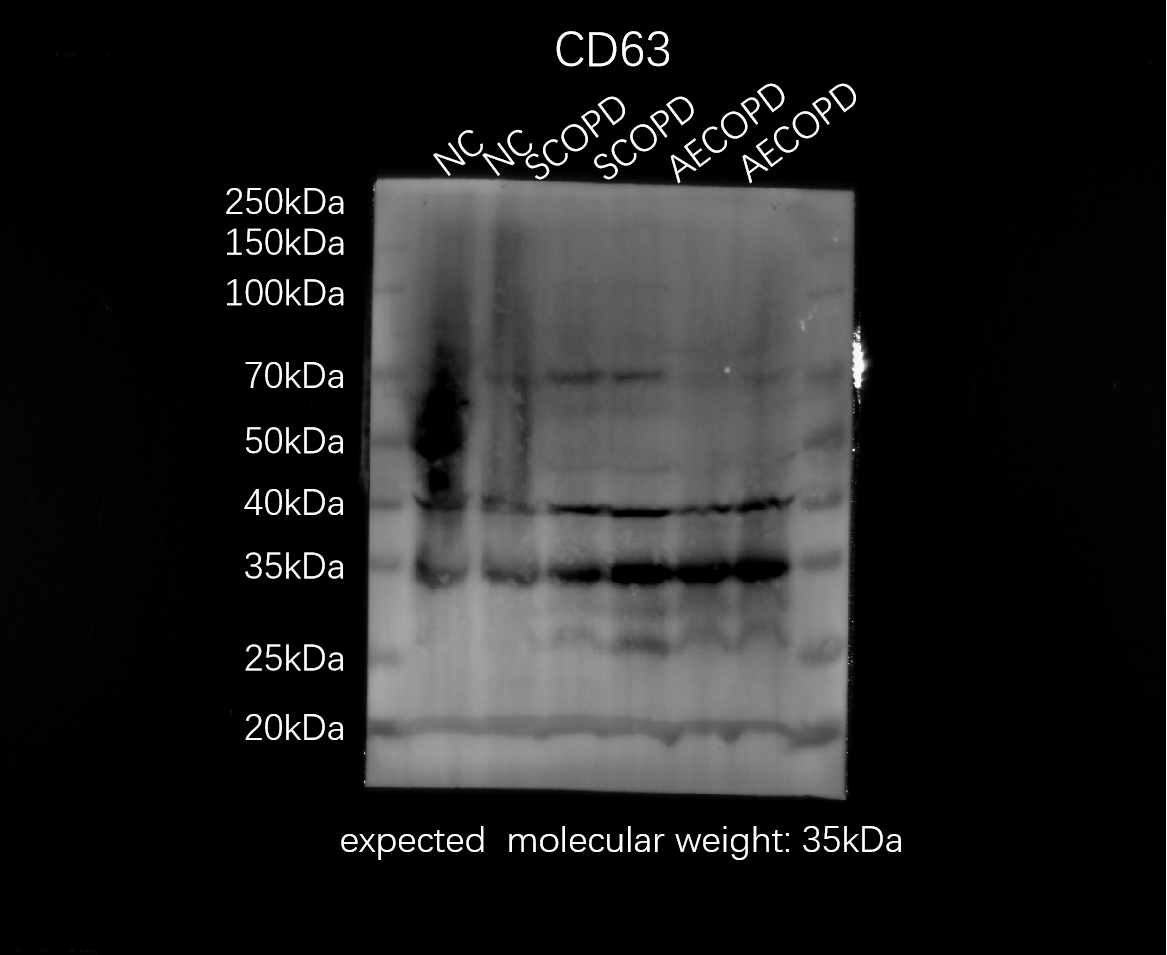


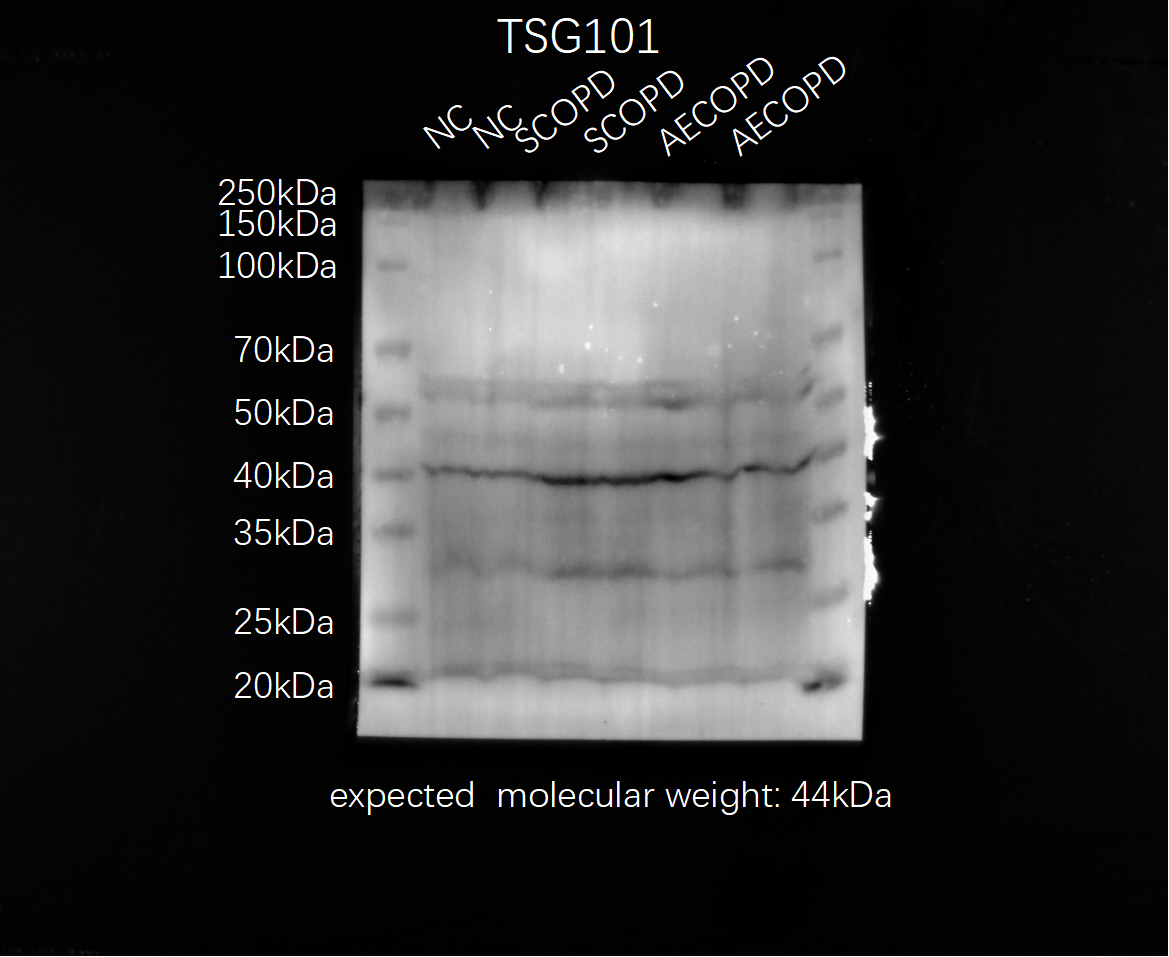


One fully gels and blots images in this time revision


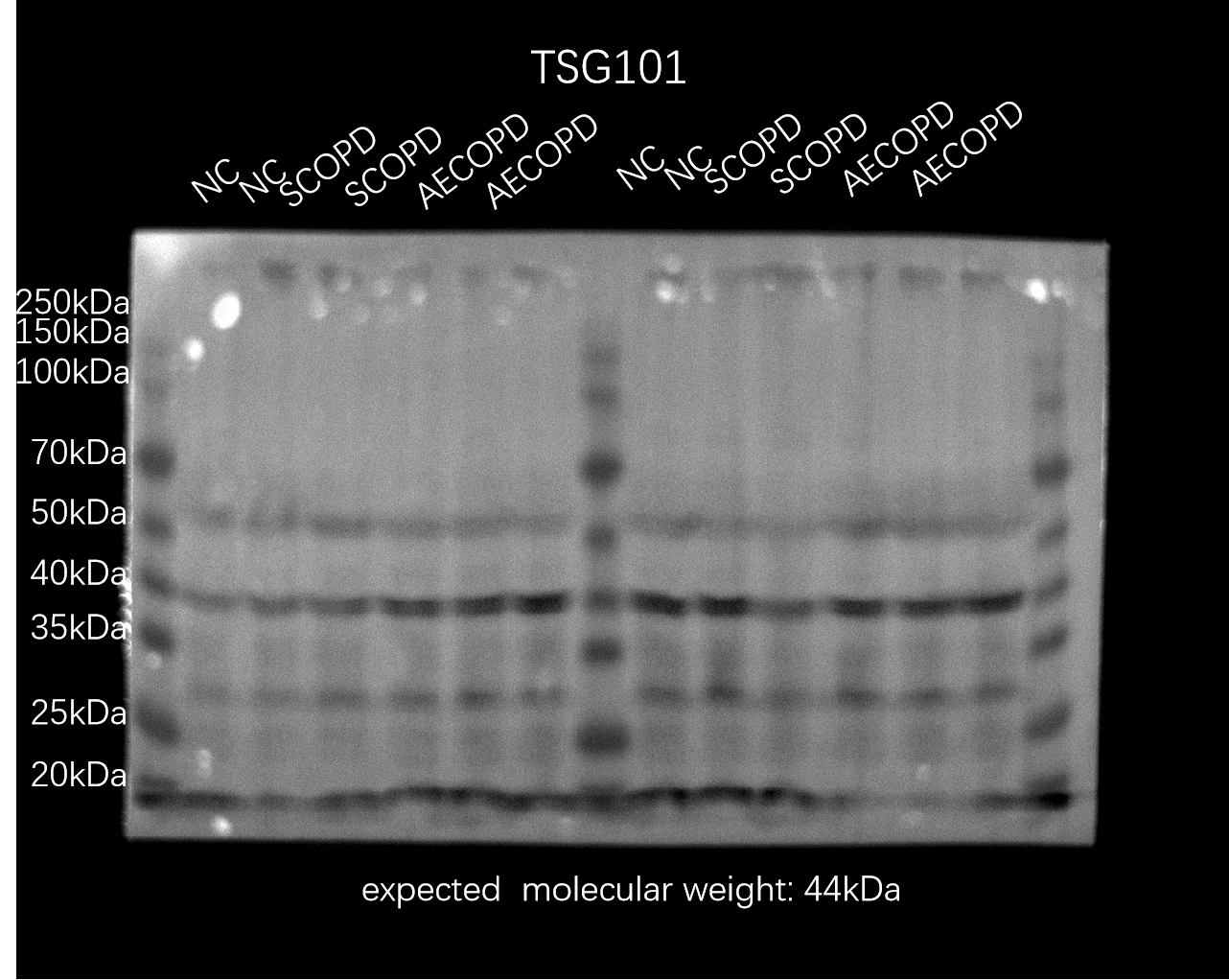


**S4.Mean diameters in serum exosomes of different groups**
